# Impact of the COVID19 pandemic on patients followed in psychiatry

**DOI:** 10.1192/j.eurpsy.2022.1253

**Published:** 2022-09-01

**Authors:** K. Razki, Y. Zgueb, A. Aissa, U. Ouali, R. Jomli

**Affiliations:** Hôpital Razi, Service Psychiatrie A, La Manouba, Tunisia

**Keywords:** Covid-19 pandemic, Impact, mental disorder, psychiatric follow-up

## Abstract

**Introduction:**

In the literature, some studies consider psychiatric patients to be vulnerable to COVID-19, in contrast to other studies that find them rather protected.

**Objectives:**

To determine the impact of the COVID 19 pandemic on patients undergoing psychiatric care.

**Methods:**

This is a descriptive and cross-sectional study that took place in the psychiatry department A at Razi hospital in Tunisia. We conducted a comparison of patient follow-up between the period of March 2018-2019 and March 2020-2021. For this we used a form including socio demographic data, data concerning the COVID-19 situation, clinical data while comparing the follow-up of patients (hospitalizations, mode of relapses, consultations in the emergency room…)

**Results:**

100 patients were included, 60% were men, mean age 44 years (+/- 11 years) [19-65 years]. Ninety-seven percent of patients had no personal history of COVID-19 infection. Comparing the pre-pandemic year (2018-2019) and the pandemic year (2020-2021), we note an increase in the rate of emergency room visits of (17.5%) as well as a relapse rate requiring hospitalization in our department in 48%, this figure was 30% in 2019. A statistically significant increase was noted for depressive and anxiety relapses (p=0.04; r=0.7). Fear of catching the virus while attending hospital facilities (17.6%), geographical isolation (17.6%), unavailability of treatment (17%) and poor insight (41.2%) were the primary causes of poor adherence.

**Conclusions:**

The patients followed in our department have presented during this COVID-19 pandemic several relapses of their psychiatric pathologies compared to the previous year.

**Disclosure:**

No significant relationships.

